# Increased binding of stroke-induced long non-coding RNAs to the transcriptional corepressors Sin3A and coREST

**DOI:** 10.1042/AN20130029

**Published:** 2013-10-23

**Authors:** Ashutosh Dharap, Courtney Pokrzywa, Raghu Vemuganti

**Affiliations:** *Department of Neurological Surgery, University of Wisconsin, Madison, WI, U.S.A.; †Theoretical Biology and Biophysics (T-6), Los Alamos National Laboratory, Los Alamos, NM, U.S.A.

**Keywords:** cerebral ischemia, corepressor, genomics, long non-coding RNA, REST, Sin3A, transcription factor, ChIP, chromatin immunoprecipitation, CMP, chromatin-modifying protein, CNS, central nervous system, Dclk1, doublecortin-like kinase 1, Fmr1, fragile X mental redardation 1, Fos, FBJ osteosarcoma oncogene, Galntrl6, UPD-*N*-acetyl-alpha-D-galactosamine: polypeptide *N*-acetylgalactosaminyltransferase-like 6, GFAP, glial fibrillary acidic protein, lncRNA, long non-coding RNA, MCAO, middle cerebral artery occlusion, REST, RE-1 silencing transcription factor, RIP, RNA immunoprecipitation

## Abstract

LncRNAs (long non-coding RNAs) are thought to play a significant role in cellular homeostasis during development and disease by interacting with CMPs (chromatin-modifying proteins). We recently showed that following transient focal ischemia, the expression of many lncRNAs was altered significantly in rat brain. We currently analyzed whether focal ischemia also alters the association of lncRNAs with the CMPs Sin3A and coREST (corepressors of the RE-1 silencing transcription factor). RIP (RNA immunoprecipitation) combined with lncRNA microarray analysis showed that 177 of the 2497 lncRNAs expressed in rat cerebral cortex showed significantly increased binding to either Sin3A or coREST following ischemia compared with sham. Of these, 26 lncRNAs enriched with Sin3A and 11 lncRNAs enriched with coREST were also up-regulated in their expressions after ischemia. A majority of the lncRNAs enriched with these CMPs were intergenic in origin. Evaluation of the expression profiles of corresponding protein-coding genes showed that their expression levels correlate with those of the lncRNAs with which they shared a common locus. This is the first study to show that stroke-induced lncRNAs might associate with CMPs to modulate the post-ischemic epigenetic landscape.

## INTRODUCTION

LncRNAs (long non-coding RNAs) are a unique class of RNAs that are >200 bp long and show specific spatiotemporal expression profiles (Batista and Chang, 2013). Perturbations in the cerebral lncRNAome were shown to exacerbate the pathophysiology of a variety of CNS (central nervous system) disorders, drug addiction and cancer (Michelhaugh et al., 2011; Pastori and Wahlestedt, 2012; Qiu et al., 2013). However, very little is known about the significance of lncRNAs after acute injuries to the CNS. We recently showed that the expression of many lncRNAs altered rapidly in rat brain following transient focal cerebral ischemia (Dharap et al., 2012). Recent studies have shown evidence of physical associations between lncRNAs and CMPs (chromatin modifying proteins) such as polycomb repressive complex 2, lysine (K)-specific demethylase, euchromatic histone-lysine N-methyltransferase 2 and heterogeneous nuclear ribonucleoprotein K (Nagano et al., 2008; Pandey et al., 2008; Khalil et al., 2009; Huarte et al., 2010; Nagano and Fraser, 2011; Rinn and Chang, 2012a). These interactions were shown to be crucial for global processes such as chromosome inactivation and lineage-specific gene repression (Nagano and Fraser, 2009; Pandey and Kanduri, 2011), as well as local events such as the p53 response to environmental insults (Huarte et al., 2010). These studies suggest that lncRNAs play an essential role in epigenetic silencing exerted by CMPs.

Recent studies showed that the neuronal REST (RE-1 silencing transcription factor) and its corepressors Sin3A and coREST were robustly activated in the rodent brain and together mediated the epigenetic silencing of several neuronal genes resulting in neuronal death after cerebral ischemia (Noh et al., 2012). REST is also known to control the expression of non-coding RNAs in brain pathologies such as Huntington's disease (Johnson et al., 2010). We currently evaluated whether stroke-responsive lncRNAs interact with Sin3A and coREST, the CMPs associated with REST (Andres et al., 1999; Grimes et al., 2000; Ballas and Mandel, 2005).

## MATERIALS AND METHODS

### Transient focal ischemia

A 1 h transient MCAO (middle cerebral artery occlusion) was induced in adult, male, spontaneously hypertensive rats (280–320 g; Charles River) under isoflurane anesthesia by the intraluminal suture method as described earlier (Dharap et al., 2012; Pandi et al., 2013). All surgical procedures were approved by the Research Animal Resources and Care Committee of the University of Wisconsin-Madison and animals were cared for in accordance with the Guide for the Care and Use of Laboratory Animals, US Department of Health and Human Services Publication number 86–23 (revised). After suturing the wound, 0.5% bupivacaine (0.25 ml) was injected along the incision to provide short duration local anesthesia. The animals were allowed to recover from anesthesia and returned to the cage with ad libitum access to food and water. During the surgery, rats were under spontaneous respiration. Rats were killed at 6 h of reperfusion and the ipsilateral cortex was dissected. Sham-operated rats served as control.

### RIP (RNA immunoprecipitation)

Cortical tissue was homogenized in 1:1 phosphate-buffered saline and nuclear isolation buffer [1.28 M sucrose; 40 mM Tris–HCl pH 7.5; 20 mM MgCl_2_; 4% (v/v) Triton X-100], centrifuged at 2750 ***g*** for 15 min and the nuclear pellet was resuspended in RIP buffer (Millipore). Resuspended nuclear fraction was mechanically sheared using a homogenizer, centrifuged at 13000 ***g*** for 10 min and the lysate was collected. Antibodies against Sin3A [polyclonal ChIP (chromatin immunoprecipitation grade) ab3479; Abcam] and coREST (polyclonal ChIP grade; 07–455; Millipore) were incubated with magnetic agarose A/G beads (Invitrogen) for 1 h and the nuclear lysates were incubated with these antibody-beads complex overnight with gentle rotation. The beads were then collected using a magnetic stand, resuspended and washed four times in RIP buffer. 20 μl of this extract was used for Western blotting to confirm Sin3A and coREST pull-down. Briefly, 20 μl of RIP lysate was electrophoresed on a denaturing PAGE, transferred onto a nitrocellulose membrane and probed using Sin3A, coREST and β-actin antibodies and the corresponding HRP (horseradish peroxidase)-tagged secondary antibodies (Cell Signaling Technology) followed by chemiluminescence detection and visualization of the bands.

### lncRNA microarray

RNA was extracted from the RIP nuclear lysate preparation using the Magna RIP kit (Millipore), linearly amplified, labeled with Cy3-dCTP, purified by RNAeasy Mini Kit (Qiagen), fragmented and hybridized to an Arraystar lncRNA expression microarray containing 9300 lncRNA probes as described previously (Dharap et al., 2012). Differentially expressed transcripts were identified by fold-change screening with a threshold of ≥2-fold. Statistically significant differences between the groups were identified by the statistical measures built in the GeneSpring based on the *t* test *P* value method with a high stringency (fold change cutoff of >2 and a probability value of <0.001 to decrease false-positives).

### Real-time PCR

The mRNA expression of Dclk1 (doublecortin-like kinase 1), GFAP (glial fibrillary acidic protein), Fmr1 (fragile X mental retardation 1); Galntrl6 (UPD-*N*-acetyl-alphaD-galactosamine:polypeptide *N*-acetylgalactosaminyltransferase-like 6); Fos (FBJ osteosarcoma oncogene) was evaluated with real-time PCR using the SYBR-Green method as described earlier using 18s rRNA as an internal control (Dharap et al., 2009). The following primer sequences (5′ to 3′) designed with Primer Express Software (Applied Biosystems) based on the GenBank numbers given in parenthesis were used for real-time PCR: Dclk1 (NM_053343): CGGCAAGTCACCAAGTCCAT and ACATCGCTCCACTGTGTCTTT; GFAP (NM_017009): GCCTCTCCCTGTCTCGAATG and CGCCTTGTTTTGCTGTTCCA; Fmr1 (NM_052804): TTGCCACCAAGTTCCCTA and AGTGGCATTAGCGATGCTGT; Galntrl6 (NM_001135756): TTGTGCGCACCAAGAAAAGG and GTGCCTCGTACCCAAAGTGA; and Fos (NM_022197): TACTACCATTCCCCAGCCGA and GCTGTCACCGTGGGGATAAA.

## RESULTS

### Association of lncRNAs with Sin3A and coREST

Cortical nuclear lysates from rats subjected to focal ischemia were IP (immunoprecipitated) for Sin3A and coREST (confirmed by Western blotting; Supplementary Figure; available at http://www.asnneuro.org/an/005/an005e124add.htm) and the precipitated RNA was subjected to lncRNA microarray analysis (RIP-chip). The RIP-chiP showed a significant increase in enrichment of 99 lncRNAs with Sin3A and 78 lncRNAs with coREST in the ischemia group compared with the sham group. Three lncRNAs showed increased binding to both Sin3A and coREST, as well as increased expression after focal ischemia compared with sham (Table 1). Twelve lncRNAs showed increased binding to both Sin3A and coREST after focal ischemia, but were not induced in their expression after stroke (Table 1). A further 23 lncRNAs that showed increased binding to Sin3A (Table 2) and eight lncRNAs that showed increased binding to coREST (Table 3) were also significantly up-regulated after focal ischemia compared with sham. A further 61 lncRNAs showed increased binding to Sin3A and 55 lncRNAs showed increased binding to coREST after focal ischemia, but none of them were induced in their expression after stroke (Supplementary Tables S1 and S2; available at http://www.asnneuro.org/an/005/an005e124add.htm).

**Table 1:** LncRNAs that showed increased binding to both Sin3A and coREST after focal ischemia All lncRNAs are confirmed to be the annotated non-coding transcripts from NCBI, ENSEMBL and UCSC genome browser. Δ fold over sham is mean fold chance (<20% S.D. in each case) in comparison with the sham group (*n*=3/group). Intragenic^a^ represents sense_exon overlap. RIP, RNA immunoprecipitation. NC, no change. Fos, FBJ osteosarcoma oncogene; Slc2a3, solute carrier family 2 (facilitated glucose transporter), member 3; Lgi3, leucine-rich repeat LGI family, member 3; Prpf4b, PRP4 pre-mRNA processing factor 4 homolog B (yeast).

	Δ fold over sham				
LncRNA	Expression	RIP Sin3A	RIP coREST	Location	Associated protein-coding gene
MRAK154943	12.91	3.42	2.56	Intergenic	
XR_005513	14.50	2.81	2.25	Intergenic	
MRAK159688	12.96	2.73	3.15	Intragenic^a^	Fos (NM_022197)
BC158675	NC	6.04	3.10	Intergenic	
MRAK013532	NC	5.59	2.55	Intergenic	
MRBC030402	NC	4.36	2.22	Intergenic	
BC094214	NC	3.98	4.28	Intergenic	
MRBC019134	NC	3.02	2.31	Intergenic	
XR_007454	NC	2.73	2.15	Intergenic	
MRBC052873	NC	2.14	2.55	Intergenic	
XR_008939	NC	2.07	2.45	Intergenic	
MRuc007jsx	NC	2.00	2.18	Intergenic	
BC063168	NC	3.14	2.93	Intragenic^a^	Slc2a3 (NM_017102)
BC158671	NC	2.48	2.10	Intragenic^a^	Lgi3 (NM_001107277)
MRAK047212	NC	2.43	2.22	Intragenic^a^	Prpf4b (NM_001011923)

**Table 2:** Stroke-induced lncRNAs that showed increased binding to Sin3A, but not to coREST All lncRNAs are confirmed to be the annotated non-coding transcripts from NCBI, ENSEMBL and UCSC genome browser. Δ fold over sham is mean fold chance (<20% S.D. in each case) in comparison with the sham group (*n*=3/group). RIP, RNA immunoprecipitation. Intragenic^a^ represents sense_exon overlap and Intragenic^b^ represents sense_intron overlap. Dclk1, doublecortin-like kinase 1; CDC91, cell division cycle 91-like 1; GFAP, glial fibrillary acidic protein.

	ΔFold over sham			
LncRNA	Expression	RIP	Location	Associated protein-coding gene
XR_008555	2.86	3.60	Intergenic	
XR_007499	6.49	3.08	Intergenic	
XR_007404	5.28	3.04	Intergenic	
XR_006148	11.70	3.02	Intergenic	
XR_007321	8.66	2.56	Intergenic	
XR_005733	8.32	2.22	Intergenic	
XR_007247	18.03	2.21	Intergenic	
XR_009083	22.07	2.13	Intergenic	
XR_009151	14.91	2.12	Intergenic	
XR_005800	15.80	2.12	Intergenic	
NR_027324	5.81	2.05	Intergenic	
XR_006778	4.96	2.02	Intergenic	
DQ266361	8.33	3.87	Intergenic	
MRAK166199	3.70	3.60	Intergenic	
XR_008295	7.93	2.88	Itergenic	
XR_008876	6.66	2.62	Intergenic	
MRAK049735	18.43	2.13	Intergenic	
XR_007101	5.74	2.08	Intergenic	
XR_007384	12.11	2.02	Intergenic	
AF030089	3.85	2.90	Intragenic^a^	Dclk1 (NM_053343)
MRAK135044	3.74	2.83	Intragenic^b^	Dclk1 (NM_053343)
AY383714	10.61	2.62	Intragenic^a^	CDC9111 (NM_181637)
EF094477	6.83	2.42	Intragenic^a^	GFAP (NM_017009)

**Table 3:** Stroke-induced lncRNAs that showed increased binding to coREST, but not to Sin3A All lncRNAs are confirmed to be the annotated noncoding transcripts from NCBI, ENSEMBL and UCSC genome browser. Δ fold over sham is mean fold chance (<20% S.D. in each case) in comparison to the sham group (*n*=3/group). RIP, RNA immunoprecipitation. Intragenic^a^ represents sense_exon overlap and Intragenic^b^ represents sense_intron overlap. Fmr1, fragile X mental redardation 1; Sh3bgrl1, SH3 domain-binding glutamic acid-rich protein like 2; Galntl1, UDP-*N*-acetyl-alpha-D-galactosamine:polypeptide *N*-acetylgalactosaminyltransferase-like 6.

	ΔFold over sham			
LncRNA	Expression	RIP	Location	Associated protein-coding gene
MRAK163011	4.19	2.15	Intergenic	
XR_008508	2.75	2.08	Intergenic	
MRAK143109	2.51	6.55	Intergenic	
XR_008791	2.81	2.22	Intergenic	
MRuc008ymd	10.45	2.19	Intergenic	
MRAK053211	6.57	6.26	Intragenic^a^	Fmr1 (NM_052804)
MRAK080604	2.21	4.46	Intragenic^a^	Sh3bgrl1 (NM_001137647)
XR_007355	7.66	3.02	Intragenic^b^	Galntl1 (NM_001135756)

Importantly, of 537 lncRNAs significantly induced (≥ 2-fold) at 6 h of reperfusion after transient MCAO, the top 17 stroke-induced lncRNAs (up-regulated from 22- to 93-fold over sham) (Dharap et al., 2012) were not enriched with either protein (Sin3A or coREST) evaluated by RIP. For the top 50 stroke-induced lncRNAs (up-regulated from 7- to 93-fold over sham), only seven showed increased enrichment with Sin3A and three showed increased enrichment with coREST. Together, this demonstrates that the enrichment of lncRNAs with Sin3A and coREST is not an artifact of an overall increase in lncRNA gene expression after stroke, but instead selective recruitment by these proteins. Interestingly, 74 and 85% of the lncRNAs enriched with Sin3A and coREST, respectively, were not significantly up-regulated following stroke suggesting that they may be present as dormant reserve pools in the healthy cortex that are subsequently targeted for recruitment by activated Sin3A and coREST following an ischemic insult.

### Genomic correlates of the Sin3A and coREST-associated lncRNAs

**Figure 1:**
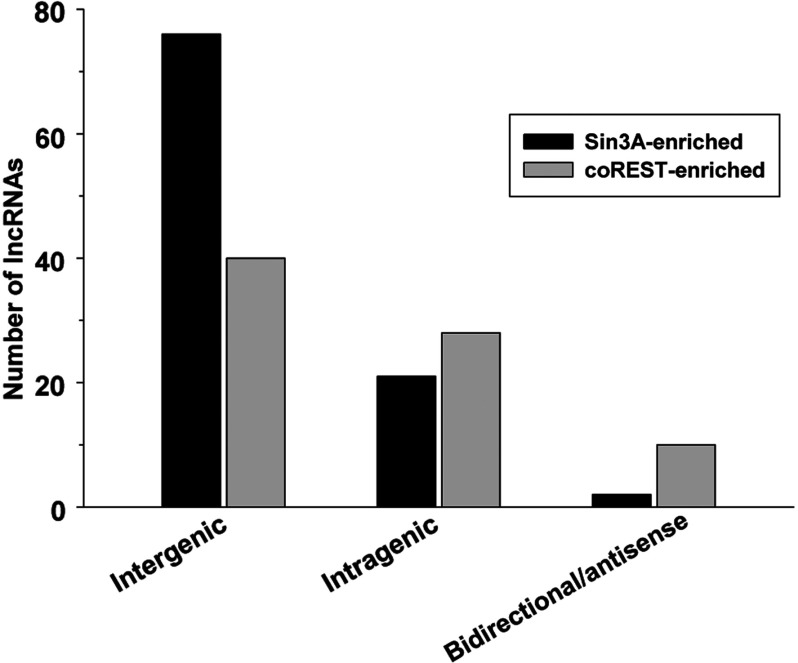
Genomic loci of the numbers of lncRNAs that showed increased binding to Sin3A and coREST after transient focal ischemia

LncRNAs are transcribed from various loci including introns of protein-coding genes (sense), antisense to protein-coding genes and intergenic stretches (Rinn and Chang, 2012b). Majority of the lncRNAs that showed significantly increased binding to Sin3A and coREST after stroke are intergenic (65%) or intragenic (sense exon or intron; 28%), while 7% are either bidirectional or antisense in origin (Figure 1).

Of the 15 lncRNAs co-enriched with both Sin3A and coREST after stroke, 11 are intergenic and 4 are intragenic (Table 1). Of the three lncRNAs that showed increased enrichment with both Sin3A and coREST as well as induced in expression after stroke, two are intergenic and one is intragenic (Table 1). The Fos locus produced an lncRNA that showed increased binding to both Sin3A and coREST (Table 1). Some Sin3A-enriched lncRNAs transcribed from intragenic loci that are particularly relevant to stroke, such as the transcription factor Fos, Dclk1 and the astrocytic activation marker GFAP (Table 2). Interestingly, the Dclk1 locus produced two lncRNAs, both of which were significantly induced after stroke and showed increased binding to Sin3A compared with sham (Table 2).

### Expression of protein-coding genes associated with the intragenic lncRNAs

**Figure 2:**
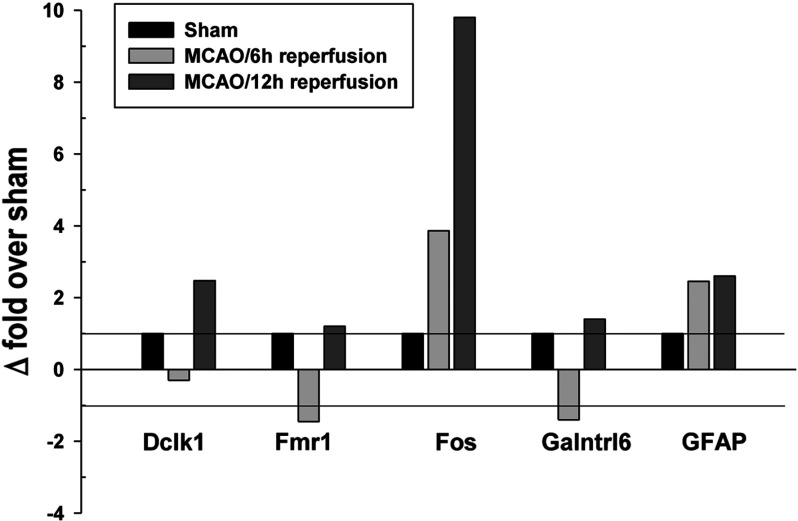
Expression levels of protein-coding RNAs originating from the same loci as intragenic lncRNAs that showed induced expression as well as increased binding to Sin3A or coREST after ischemia

The observation that several Sin3A and coREST associated lncRNAs originated from intragenic loci suggested that their expression might be associated with that of the corresponding protein-coding transcripts. Hence, we analyzed the post-ischemic expression of the protein-coding genes Dclk1, GFAP, Fmr1, Galntrl1 and Fos associated with the intragenic lncRNAs that were up-regulated in their expression as well as association to Sin3A and/or coREST. Of these, Fos was up-regulated by 3.8-fold at 6 h reperfusion and 9.8-fold at 12 h reperfusion compared with sham (Figure 2). GFAP was up-regulated by 2.5-fold at 6 h and 2.6-fold at 12 h reperfusion compared with sham (Figure 2). Dclk1 showed no significant change at 6 h but was up-regulated by 2.5-fold at 12 h reperfusion compared with sham (Figure 2). Whereas, Fmr1 and Galntrl6 showed no significant change over sham at any reperfusion time after transient MCAO (Figure 2).

## DISCUSSION

The mammalian genome transcribes numerous lncRNAs that show evolutionary conservation and are expressed in a stage and cell specific manner (Mercer and Mattick, 2013; Sabin et al., 2013). Recent studies suggested that altered levels and/or functionality of lncRNAs play a role in various pathologies including cancer, Alzheimer's disease and myocardial infarction (Pastori and Wahlestedt, 2012; Ounzain et al., 2013; Qiu et al., 2013).

A major function attributed to lncRNAs is to bind with CMPs and modulate their transcriptional activity (Rinn and Chang, 2012a). Hence, a change in the expression profiles of lncRNAs under pathological conditions might significantly influence the functionality of CMPs. We recently showed that stroke alters the expression profiles of lncRNAs in a rapid and significant manner in rodent brain (Dharap et al., 2012). To understand the implications of the altered lncRNAome in post-stroke pathophysiology, we currently evaluate whether ischemia promotes the association of lncRNAs with the CMPs that act as corepressors of the transcription factor REST. Recent studies showed that REST up-regulation promotes post-ischemic neuronal death (Noh et al., 2012; Pandi et al., 2013). REST is a transcription factor that plays a significant role in the phenotypic maturation of neurons by silencing neural genes in the non-neural cells of CNS during development. REST expression in the normal, adult brain is known to be low. Sin3A and coREST are essential corepressors of REST that bind to its opposite terminals and recruit other silencing factors to the target loci (Ballas and Mandel, 2005). Our results show that the physical association of many lncRNAs with Sin3A and coREST increase significantly following stroke. Although lncRNA expression and binding to CMPs are independent events, several lncRNAs that showed increased binding to Sin3A and coREST also showed increased expression following focal ischemia. This suggests that the Sin3A- and coREST-enriched lncRNAs induced after stroke might play important roles in modifying the post-ischemic epigenetic landscape by modulating the REST-mediated gene silencing.

Previous work exploring the binding of various CMPs to lncRNAs showed that each protein has a distinct lncRNA signature with marginal overlap with other related CMPs (Khalil et al., 2009). Our data support this by showing that a majority of the stroke-induced lncRNAs were bound to Sin3A or coREST with only three common lncRNAs that bound to both. Since Sin3A and coREST bind opposite termini of REST, these overlapping lncRNAs might serve as a scaffold to recruit Sin3A and coREST to the REST complex and/or facilitate the recruitment of other proteins such as histone deacetylases that are common to both corepressors (Ballas and Mandel, 2005; Roopra et al., 2012).

Most of the lncRNAs that showed increased binding to Sin3A and coREST are intergenic. However, some are also intragenic and were mapped to the loci of protein-coding genes such as Fos, Dclk1, GFAP and Fmr1. The impact of the intragenic lncRNAs on the expression of the corresponding protein-coding transcripts is unclear. LncRNAs can silence or activate the expression of the associated genes *cis* or in *trans* (Batista and Chang, 2013). The expression of lncRNA MRAK159688 that originates from an exon of Fos was observed to be up-regulated by ~13-fold after focal ischemia. RIP data showed that the enrichment of MRAK159688 with both Sin3A and coREST also increased after focal ischemia by ~3-fold. We observed that Fos expression was also induced significantly after ischemia. Although MRAK159688 is not transcribed by Fos *per se*, their co-expression from the same locus may position the lncRNA to serve as an upstream regulator or downstream effector of Fos probably via REST and its corepressors. A similar functional association was previously shown for lincRNA-p21 and p53. The lincRNA-p21 induced by p53 was shown to associate with a p53 complex to repress the expression of p53 target genes (Huarte et al., 2010). AF030089 and MRAK135044 are stroke-induced lncRNAs that originate from a sense exon and sense intron, respectively, of the *Dclk1* gene. Both lncRNAs showed identical expression levels (distinct probes incorporating the sequence variation were used to detect these two lncRNAs) and were significantly enriched with Sin3A. This suggests that these two lncRNAs might control Dclk1 function via Sin3A in the post-ischemic cortex. The lncRNA EF094477 is also intragenic from GFAP gene locus. We observed that the expression and binding to Sin3A of EF094477 were induced after ischemia by 6.8- and 2.4-fold, respectively, and GFAP expression was also induced concomitantly. Not all lncRNA/protein-coding pairs from the same loci will be co-regulated. For example, expression of Dclk1 and Galntrl1 was not altered although the lncRNAs associated with their gene loci (AF030089, MRAK1335044 and XR007355) were up-regulated in their expression as well as binding to Sin3A/coREST. However, our data indicate that the stroke-induced lncRNAs expressed from intragenic loci does not exhibit a *cis* inhibitory effect. Instead, these lncRNAs might either induce or serve to maintain the expression of the protein-coding transcripts at a normal level by activating the epigenetic signatures as demonstrated previously (Huang et al., 2010). Furthermore, some lncRNAs showed coexpression with their corresponding protein-coding genes while others did not. Hence, it is possible that intragenic lncRNAs may be transcribed differentially from the same transcription start sites as that of the corresponding protein-coding genes or via novel internal start sites independent of the protein-coding gene unit.

In summary, this is the first study to demonstrate significantly increased association between ischemia- induced lncRNAs with CMPs Sin3A and coREST. While we studied only two CMPs, there might be others that are active in the post-ischemic brain and may bind to ischemia-induced lncRNAs to form unique ribonucleoprotein functional complexes. Such interactions might be functionally significant for the ischemic pathophysiology.

## Online data

Supplementary data
