# Supplementary material for: Increased binding of stroke-induced long non-coding RNAs to the transcriptional corepressors Sin3A and coREST
Source: ASN Neuro. 2013 Oct 23;5(4):e00124. doi: 10.1042/AN20130029 (PMC3806319; doi:10.1042/AN20130029)
Supplement: Supplementary data [file an005e124add.pdf]

ASN NEURO RESEARCH ARTICLE

ASN NEURO 5(4):art:e00124.doi:10.1042/AN20130029

OPEN ACCESS

Increased binding of stroke-induced long non-coding RNAs to the transcriptional corepressors Sin3A and coREST

Ashutosh Dharap*†[1], Courtney Pokrzywa* and Raghu Vemuganti*[1]

*Department of Neurological Surgery, University of Wisconsin, Madison, WI, U.S.A.

†Theoretical Biology and Biophysics (T-6), Los Alamos National Laboratory, Los Alamos, NM, U.S.A.

SUPPLEMENTARY DATA

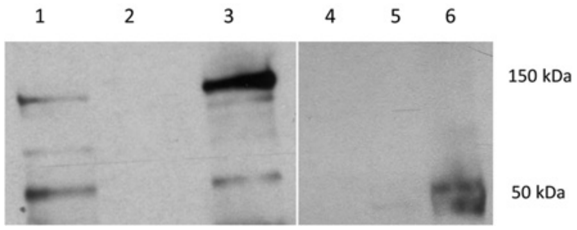

Figure S1 Immunoblotting of Sin3A and coREST from immunoprecipitated nuclear lysates

Antibodies that were used to immunoprecipitate SinA and coREST proteins from nuclear lysates were used to probe for the respective proteins by Western blotting. The Sin3A band is visible at the 150 kDa marker and coREST is visible at the 50 kDa marker. Lane 1 – Sin3A input; Lane 2- Negative IgG RIP; Lane 3 – Sin3A RIP; Lane 4 – coREST input; Lane 5- Negative IgG RIP; Lane 6 – coREST RIP.

[1]Correspondence may be addressed to either of these authors (email vemuganti@neurosurgery.wisc.edu or Dharap.wisc@gmail.com).

© 2013 The Author(s) This is an Open Access article distributed under the terms of the Creative Commons Attribution Licence (CC-BY) (http://creativecommons.org/licenses/by/3.0/) which permits unrestricted use, distribution and reproduction in any medium, provided the original work is properly cited.

Table S1 LncRNAs that showed increased binding to Sin3A, but not induced in their expression after ischemia

All lncRNAs are confirmed to be the annotated noncoding transcripts from NCBI, ENSEMBL and UCSC genome browser. Δ fold over sham is mean fold chance (<20% S.D. in each case) in comparison with the sham group ($n = 3$/group). RIP, RNA immunoprecipitation. Intragenic[a] represents sense_exon overlap and Intragenic[b] represents sense_intron overlap.

LncRNA	ΔFold over sham	Location	Associated protein-coding gene
MRuc007pas	5.92	Intergenic	
S66184	4.10	Intergenic	
DQ266361	3.86	Intergenic	
MRAK166199	3.60	Intergenic	
XR_007305	3.47	Intergenic	
AF050659	3.02	Intergenic	
DQ359102	2.98	Intergenic	
S77494	2.96	Intergenic	
XR_005868	2.91	Intergenic	
BC158811	2.80	Intergenic	
MRAK013736	2.66	Intergenic	
BC085903	2.66	Intergenic	
MRuc009dpe	2.62	Intergenic	
BC166474	2.61	Intergenic	
BC161988	2.60	Intergenic	
XR_007040	2.60	Intergenic	
BC166932	2.54	Intergenic	
XR_006309	2.51	Intergenic	
MRAK019982	2.49	Intergenic	
XR_005636	2.46	Intergenic	
MRAK017754	2.43	Intergenic	
XR_005617	2.42	Intergenic	
AF545831	2.39	Intergenic	
S74342	2.38	Intergenic	
XR_007959	2.34	Intergenic	
MRAK164163	2.32	Intergenic	
BC100074	2.29	Intergenic	
BC090353	2.28	Intergenic	
MRAK012211	2.28	Intergenic	
MRAK017269	2.25	Intergenic	
XR_009582	2.23	Intergenic	
BC166798	2.21	Intergenic	
XR_006612	2.18	Intergenic	
XR_008036	2.18	Intergenic	
XR_007929	2.17	Intergenic	
XR_005433	2.16	Intergenic	
XR_008741	2.15	Intergenic	
BC064030	2.12	Intergenic	
MRAK131843	2.11	Intergenic	
BC166935	2.11	Intergenic	
XR_009451	2.10	Intergenic	
MRuc007kvk	2.09	Intergenic	
BC088349	2.09	Intergenic	
MRAK164927	2.04	Intergenic	
MRAK040264	2.01	Intergenic	
XR_006768	2.00	Intergenic	
MRAK082557	4.06	Intragenic[a]	Ncam1 (NM_031521)
MRAK005234	3.68	Intragenic[a]	Rbm39 (NM_001013207)
BC168954	3.47	Intragenic[a]	Spag9 (NM_001108290)
BC088258	2.90	Intragenic[a]	RGD1303066 (NM_212498)

© 2013 The Author(s) This is an Open Access article distributed under the terms of the Creative Commons Attribution Licence (CC-BY) (http://creativecommons.org/licenses/by/3.0/) which permits unrestricted use, distribution and reproduction in any medium, provided the original work is properly cited.

Table S1 Continued

LncRNA	ΔFold over sham	Location	Associated protein-coding gene
MRAK038961	2.66	Intragenic[a]	Prkcbp1 (NM_001100838)
XR_007265	2.63	Intragenic[a]	Polr2l (NM_001143911)
MRAK043151	2.21	Intragenic[a]	CD47 (NM_019195)
MRAK136334	2.16	Intragenic[a]	Eny2 (NM_001130580)
BC167004	2.12	Intragenic[a]	Mapk1ip1l (NM_001108373)
X05699	2.00	Intragenic[a]	S100a6 (NM_053485)
MRAK167711	2.00	Intragenic[a]	Slc16a1 (NM_012716)
BC169038	2.13	Intragenic[a]	unknown
MRAK080234	2.55	Intragenic[b]	Lamp2 (NM_017068)
XR_007265	2.63	Antisense_exon	Tspan4 (NM_001013070)
XR_008769	2.37	Bidirection	Tssk6 (NM_001106078)

Table S2 LncRNAs that showed increased binding to coREST, but not increased expression following focal ischemia

All lncRNAs are confirmed to be the annotated non-coding transcripts from NCBI, ENSEMBL and UCSC genome browser. Δ fold over sham is mean fold chance (<20% S.D. in each case) in comparison with the sham group ($n = 3$/group). RIP, RNA immunoprecipitation. Intragenic[a] represents sense_exon overlap and Intragenic[b] represents sense_intron overlap.

LncRNA	ΔFold over sham	Location	Associated protein-coding gene
BC099234	8.60	Intergenic	
AF322224	7.62	Intergenic	
MRAK143109	6.55	Intergenic	
MRAK035295	5.30	Intergenic	
MRAK164152	5.11	Intergenic	
MRuc007pap	4.82	Intergenic	
MRAK046848	4.34	Intergenic	
XR_005561	4.03	Intergenic	
BC097997	3.06	Intergenic	
MRNR_002452	2.97	Intergenic	
MRuc009pdp	2.80	Intergenic	
MRAK050417	2.73	Intergenic	
MRuc009mni	2.40	Intergenic	
MRAK021346	2.34	Intergenic	
XR_008147	2.33	Intergenic	
AF435963	2.28	Intergenic	
XR_008791	2.22	Intergenic	
MRuc008ymd	2.19	Intergenic	
BC168162	2.18	Intergenic	
AJ437403	2.16	Intergenic	
L22653	2.13	Intergenic	
XR_009193	2.07	Intergenic	
AF267752	2.02	Intergenic	
MRAK086453	2.00	Intergenic	
MRAK028347	19.20	Intragenic[a]	Rbm4b (NM_001007014)
MRAK045560	14.70	Intragenic[a]	Disp2 (NM_001107759)
uc.63-	5.44	Intragenic[b]	Xpo1 (NM_053490)
MRAK083513	5.12	Intragenic[a]	Recql5 (NM_001105853)
AY007691	4.75	Intragenic[a]	Vcan variants 1, 2, 3 and 4 (NM_001170558, NM_053663, NM_001170560, NM_001170559)
MRAK014035	3.48	Intragenic[a]	Tmem256 (NM_001170549)
MRAK010087	3.33	Intragenic[a]	RGD1562161 (NM_001127568)

© 2013 The Author(s) This is an Open Access article distributed under the terms of the Creative Commons Attribution Licence (CC-BY) (http://creativecommons.org/licenses/by/3.0/) which permits unrestricted use, distribution and reproduction in any medium, provided the original work is properly cited.

Table S2 Continued

LncRNA	ΔFold over sham	Location	Associated protein-coding gene
BC063168	2.93	Intragenic[a]	Slc2a3 (NM_017102)
EF688596	2.88	Intragenic[a]	Nol6 (NM_001107927)
S81002	2.42	Intragenic[b]	MAP2 (NM_013066)
EF613276	2.36	Intragenic[a]	Dvl2 (NM_001172056)
MRuc008vjw	2.32	Intragenic[a]	Eif4g3 (NM_001106693)
MRAK014638	2.35	Intragenic[a]	Spag3 (NM_001108290)
BC161977	2.31	Intragenic[a]	RGD1566127 (NM_001109156)
MRAK050935	2.28	Intragenic[a]	Gab1 (NM_001108444)
MRuc009ktv	2.24	Intragenic[a]	Ccl25 (NM_001037203)
BC111077	2.13	Intragenic[a]	Fam129c (NM_001100908)
BC169100	2.13	Intragenic[a]	Gcn1l1 (NM_001168664)
MRuc008sut	2.09	Intragenic[a]	Nr4a3 (NM_031628)
			Tmem14c variant 2 (NM_001135169)
DQ832324	2.05	Intragenic[a]	Ednra (NM_012550)
X93219	2.04	Intragenic[a]	Adora3 (NM_012896)
MRuc007nzw	2.64	Antisense_exon	Arg2 (NM_019168)
BC099096	2.20	Antisense_exon	Tmem14c variant 1 (NM_134395)
BC158613	3.56	Antisense_exon	LOC64038 (NM_022271)
MRAK083513	5.12	Antisense_intron	Smim5 (NM_001163002)
MRAK011616	3.65	Antisense_intron	C4b (NM_031504)
uc.308-	2.12	Antisense_intron	Btrc (NM_001007148)
MRAK041468	2.31	Antisense_intron	Gpc3 (NM_012774)
MRAK083513	5.12	Bidirection	Sap30bp (NM_001108305)
MRAK011616	3.65	Bidirection	Ppt2 (NM_019367)
MRuc009ktv	2.24	Bidirection	Elavl1 (NM_001108848)

© 2013 The Author(s) This is an Open Access article distributed under the terms of the Creative Commons Attribution Licence (CC-BY) (http://creativecommons.org/licenses/by/3.0/) which permits unrestricted use, distribution and reproduction in any medium, provided the original work is properly cited.
